# Editorial: Nutritional and physical activity strategies to boost immunity, antioxidant status and health, Volume II

**DOI:** 10.3389/fphys.2022.1050549

**Published:** 2022-11-01

**Authors:** Mallikarjuna Korivi, Arifullah Mohammed, Weibing Ye, Veeranjaneya Reddy Lebaka

**Affiliations:** ^1^ Exercise and Metabolism Research Center, College of Physical Education and Health Sciences, Zhejiang Normal University, Jinhua, China; ^2^ Department of Agriculture, Faculty of Agro-Based Industry, Universiti Malaysia Kelantan, Jeli, Kelantan, Malaysia; ^3^ Department of Microbiology, Yogi Vemana University, Kadapa, India

**Keywords:** physical exercise, alternative therapeutic, inflammation, food supplement, antioxidant status

In addition to the existing knowledge, here we have emphasized the recent developments on the role of nutritional supplements and physical activity in improving immunity, inflammatory response, redox signaling, and health status. This Research Topic includes research articles, reviews, and meta-analyses, which demonstrate the beneficial effects of physical activity with or without a combination of nutritional interventions among healthy individuals and patients. A comprehensive review by Shao et al. highlights that the intake of proper nutrients together with physical activity can improve the energy balance, immune system, and functional ability of various systems. This study enlightens that the incorporation of various fruits and vegetables, whole grains, proteins, and probiotics are vital for sustaining overall health. In addition to physical activity, supplementation with certain probiotics, plant-derived compounds, and functional foods may improve the immune system and prevent diseases (Shao et al.). Plant-based natural products are widely used in the treatment of several inflammatory diseases, including rheumatoid arthritis (RA). This chronic inflammatory and autoimmune disease is represented by impaired inflammatory response, pain, peripheral joint swelling, and articular cartilage damage. A study by Majeed et al. demonstrates that extracts of the *Boswellia serrata* plant reduce clinical signs of joint swelling and preserve the matrix proteins by inhibiting the hydrolyzing enzymes. This is further accompanied by decreased oxidative stress and pro-inflammatory mediators after *B. serrata* treatment (Majeed et al.). In line with this, irisin is a novel molecule that has been investigated for the regulation of metabolic syndrome-induced male infertility. A review by Sengupta et al. states that irisin can reverse the adversities of metabolic syndrome-induced male fertility disruptions and ameliorates spermatogenesis and steroidogenesis. This could be possibly due to its direct and/or indirect beneficial effects of altering insulin resistance, oxidative stress, inflammation, and testicular functions (Sengupta et al.).

Regardless of nutritional patterns or exercise behavior, negative lifestyle behavior (i.e. the consumption of alcohol) has been associated with the occurrence of various diseases, including alcoholic fatty liver. Excessive alcohol consumption is frequently associated with anxiety and depression and neuropeptide-Y (NPY) is associated with the positive or negative emotions of a healthy adult (Chen et al.). A meta-analysis was conducted to evaluate the role of neuropeptide-Y (NPY) in the development of alcoholism. This large-scale meta-analysis concludes that NPY rs16139 polymorphism is not associated with alcoholism (Chen et al.). On the other hand, COVID-19 patients after the early phase of discharge may also experience some negative emotions, such as anxiety or depression. Such mental health in patients further leads to a decrease in the quality of life, which was particularly seen among female patients (Hu et al.). Therefore, the implementation of gender-specific rehabilitation programs and other interventions is necessary to improve the psychological status, quality of life, and physical function among COVID-19 discharged patients (Hu et al.).

The beneficial effects of exercise and nutritional interventions could be influenced by various factors, including characteristics of participants (age, gender, health status, and body weight) and exercise variables (frequency, intensity, type, and duration). To address this issue, a systematic review and meta-analysis are conducted on patients with non-alcoholic fatty liver disease (NAFLD) and concludes that exercise intervention can decrease serum transaminases in patients. However, the beneficial effects are associated with patients’ age, not with gender, body mass index, and exercise variables. To be specific, young NAFLD patients are more highly responsive to the exercise intervention than older patients (Hong et al.). It is well-known that carbohydrate is the primary energy source for endurance exercise, and supplementation of carbohydrate with or without protein could influence performance and post-exercise recovery. A study by Tan and others Tan et al. demonstrates that consumption of a pre-exercise carbohydrate drink containing natural soy or whey protein had no immediate benefit on performance improvement and antioxidant status. However, fatigue recovery appears to be improved in endurance performance on day 2 after supplementation (Tan et al.), which indicates the importance of dietary supplements during post-exercise recovery.

Practicing traditional exercise programs, such as yoga, Tai-chi, Qigong and square dance has long been claimed to promote or maintain psychological and physical health. In a comparative study, older adults with either a Tai-chi or square dance practice show better immune function, physical health, and life satisfaction than the sedentary control adults. However, the Tai-chi practitioners show better physical health and immune function outcomes than the square dance practitioners. These findings suggest that Tai-chi exercise could be a practical strategy to promote overall health in Chinese older adults (Su and Zhao.). Local government attention and reforms on exercise participation and nutrition are beneficial in improving public health. A study from China validates the influential role of government attention on the Chinese population’s nutrition, health, and exercise (Zhang et al.). The findings reveal that increased implementation of government policies related to nutrition, exercise, and health has led to promoting the level of nutrition, exercise, and health of Chinese nationals. Furthermore, the total production of various food types, dietary structure, number of medical institutions, number of sports venues, and average life expectancy of the population has been constantly increasing since the reforms implemented by the Chinese government (Zhang et al.).

It is indicated that dietary habits and exercise interventions are playing a key role in improving immune function, inflammatory response, antioxidant status, and psychological well-being among adults. Nevertheless, to achieve the maximum benefits without adverse effects, the intervention programs should be tailored according to the physical status, age, and health condition of an individual.

